# Case of Severe Pain Following Labor

**Published:** 1848

**Authors:** 


### ARTICLE IV.

### *Case of Severe Pain Following Labor.*

Dr. Jas. M. Harnett, of Cochran's Grove, Shelby Co. Ill., sends us the following history of a case in which severe pain following delivery was not relieved until 550 gtts. of laudanum had been administered.

Mrs. S.---aged 35, being delivered of her first, a large healthy child, after several hours of severe labor was taken with severe pain at the upper part of the sacrum, which required for its relief the above named amount of laudanum.

There was nothing remarkable in the case during labor or convalescence; he finds it difficult therefore, to account for its appearance, and wishes us to give our opinion as to its cause, and asks if the administration of so large a dose was justifiable under the circumstances?

In answer we will state that in our opinion the pain was neuralgic caused by irritation of the branches of the sacral nerves, and that we consider the administration of opium justafiable in such cases in doses sufficient to give relief, even if twice the quantity given in the above case should be required, especially when we consider that opium as well as other medicines is liable to adulteration, and that therefore, the strength of any given quantity must be determined either by watching its effects, or by chemical analysis.

